# Prediction of highly expressed genes in microbes based on chromatin accessibility

**DOI:** 10.1186/1471-2199-8-11

**Published:** 2007-02-13

**Authors:** Hanni Willenbrock, David W Ussery

**Affiliations:** 1 Center for Biological Sequence Analysis, BioCentrum, Technical University of Denmark, DK-2800 Kgs. Lyngby, Denmark

## Abstract

**Background:**

It is well known that gene expression is dependent on chromatin structure in eukaryotes and it is likely that chromatin can play a role in bacterial gene expression as well. Here, we use a nucleosomal position preference measure of anisotropic DNA flexibility to predict highly expressed genes in microbial genomes. We compare these predictions with those based on codon adaptation index (CAI) values, and also with experimental data for 6 different microbial genomes, with a particular interest in experimental data from Escherichia coli. Moreover, position preference is examined further in 328 sequenced microbial genomes.

**Results:**

We find that absolute gene expression levels are correlated with the position preference in many microbial genomes. It is postulated that in these regions, the DNA may be more accessible to the transcriptional machinery. Moreover, ribosomal proteins and ribosomal RNA are encoded by DNA having significantly lower position preference values than other genes in fast-replicating microbes.

**Conclusion:**

This insight into DNA structure-dependent gene expression in microbes may be exploited for predicting the expression of non-translated genes such as non-coding RNAs that may not be predicted by any of the conventional codon usage bias approaches.

## Background

Transcription of DNA is highly influenced by DNA bending and flexibility. These structural properties are dependent on the base sequence [1], which in turn, is reflective of, or may influence the codon usage – also important in determining the relative expression of a given gene. Prediction of highly expressed genes and elucidation of the physical and biological properties of highly expressed genes has been addressed by a number of studies [2-4].

The translational 'codon adaptation index' (CAI) is highly correlated with the expression level in fast growing bacteria [5]. It is based on the finding that highly expressed genes almost exclusively use those codons of abundant tRNAs in *Escherichia coli *and budding yeast [4]. Consequently for any sequenced bacterial genome, a codon bias signature can be deduced that is most likely to be efficient for translation. This bias is used to derive codon adaptation indices for all genes for a given organism, where high CAI values correspond to genes most likely to be highly expressed.

However, using CAI, one is only able to predict highly expressed proteins (translated genes) since this measure is based on codon usage bias. Unfortunately, this method cannot consider tRNAs, ribosomal RNAs, and other non-coding RNAs. Moreover, for organisms with low translational bias – typically slow growing organisms – CAI is a less effective predictor of highly expressed genes [6]. Furthermore, effective usage of CAI requires the identification of a representative subset of highly expressed genes in an organism on which the codon bias is based. While relatively good subsets may be found by simple BLAST searches [7] for organisms closely related to well-characterized model organisms such as Yeast and *E. coli*, it is more difficult for more distant microbes such as archaeabacteria.

On a more global scale, gene expression may be regulated from specific promoters that are sensitive to DNA superhelicity. That is, supercoiling may regulate gene expression at a genome-wide level [8,9]. In this way, an organism may react rapidly to changes in growth and nutritional states as well as environmental conditions since DNA superhelicity varies with the cellular energy charge, which, for example, differs in log phase versus stationary phase or is influenced by environmental factors such as temperature or osmotic stress [10]. Such structural elements appear to be clustered around the chromosome in so-called topological domains [8,11,12].

The 'position preference' measure is a DNA structural measure that was originally derived for eukaryotes using chicken DNA and is a trinucleotide model of nucleosome positioning patterns. It reflects the preference of a given trinucleotide for being found in a region where the DNA minor groove faces either towards or away from the nucleosome histone core [13]. Here, we use a minor modification of the original nucleosomal positioning trinucleotide scale where absolute values reflect the magnitude of position preference [14]. Thus, high absolute position preference reflects a high preference for nucleosomes, while low absolute position preferences reflect trinucleotides which tend to exclude nucleosomes. On the one hand, this only makes sense in eukaryotes since prokaryotes do not have nucleosomes. However, prokaryotes also have chromatin, and the DNA is compacted to similar levels (i.e., more than 1000x) in both prokaryotes and eukaryotes. The position preference value is also a measure of anisotropic DNA flexibility of certain trinucleotides, which can either favor nucleosome positioning ("high position preference") or tend not to be found in sequences wrapped around nucleosomes. Consequently, the 'position preference' measure also describes a more general structural property of DNA – that is, how easily can it be wrapped around chromatin proteins. As a result, position preference has been used previously to show structural characteristics in prokaryotic genomes [14,15]. For example, a cluster analysis of various structural properties including position preference, identified groups of genes that contained all the ribosomal RNAs and a majority of the ribosomal proteins from *Escherichia coli *[15]. These genes were characterized by higher than average DNaseI sensitivity [16] and low position preference, indicating regions of DNA not easily condensed by chromatin. Since the ribosomal genes are among the most highly expressed in actively dividing *E. coli *cells, it was hypothesized that their common structural features may play a role in regulating expression and that there exists a correlation between low position preference values and highly expressed genes [17]. This makes sense because regions of DNA that are not condensed into chromatin are more accessible to the RNA polymerase. Consequently, transcription is thought to be governed by 'effective' superhelicity, where topoisomerases, the transcription machinery and chromatin proteins compete for available supercoils [18].

Here, we use the position preference (PP) measure for the prediction of highly expressed genes in 6 sequenced microbial genomes with a particular interest in the model organism *E. coli*. The predictions are compared to experimental data as well as predictions by CAI and we thereby demonstrate that the position preference measure is a useful measure for prediction of highly expressed non-translated genes. We have extended this analysis by examining position preference values of genes in 328 sequenced microbial genomes. By characterizing the functional categories of genes predicted to be highly expressed, we find that these categories are independent of phylogeny but rather reflect the ecology of the organism, such as pathogens or extremophiles.

## Results and discussion

### Whole genome E. coli Atlas

**Figure 1:**
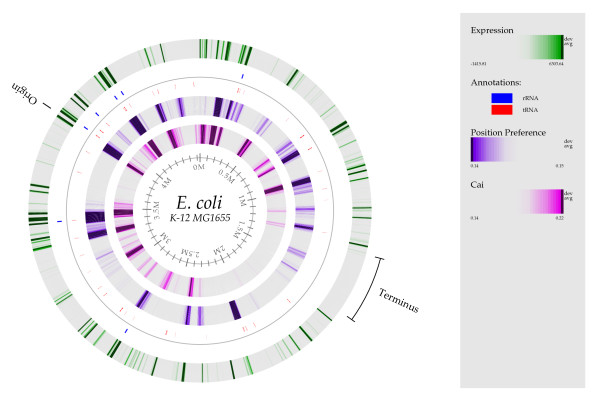
**Atlas illustration of the *E. coli *genome**. The atlas illustrates CAI values, position preference values and microarray gene expression values for *E. coli*. The average position preference values and gene expression values are illustrated for all genes at their genomic position. The CAI values can only be calculated for protein encoding genes and are therefore only illustrated for these. The range of all three color scales corresponds to ± 1.5 standard deviations.

On a chromosomal level, we observe that, for the *E. coli *K-12 genome, both CAI values and position preference values predict the same general regions of highly expressed genes, and indeed these regions correspond well with the experimental expression values (Figure 1). However, there are two regions where CAI and position preference differ significantly – the first is around 0.45 Mbp, where a cluster of highly expressed genes is predicted by CAI but not by position preference. This region contains the *cyoA*-*cyoE *genes involved in aerobic energy metabolism and they predominate during growth at high aeration. The second different region is towards the bottom of the atlas, around 2 Mbp, where there is a region with low position preference, but close to average CAI values. This region contains genes from the *flu *loci, which can be highly expressed under the appropriate environmental conditions [19]. Moreover, in Figure 1, the location of rRNAs and tRNAs are also indicated. The expression level for these genes cannot be predicted by CAI since they are not translated. The position preference measure predicts high expression within several regions concentrated in tRNAs or rRNAs. According to experimentally determined expression levels, many of these regions are also rich in highly expressed genes. Consequently, our data show that not only is the gene expression regulated by DNA structural elements as demonstrated previously [9,18], but the highly transcribed regions are also correlated with regions of the chromosome having low position preference.

### Ribosomal proteins and non-translated RNA

Ribosomal proteins are often highly expressed and demonstrate high codon usage bias in terms of high CAI values, at least in fast replicating microbes. Consequently, for these microbes, we expect a similar correlation between low position preference and high gene expression level.

**Figure 2:**
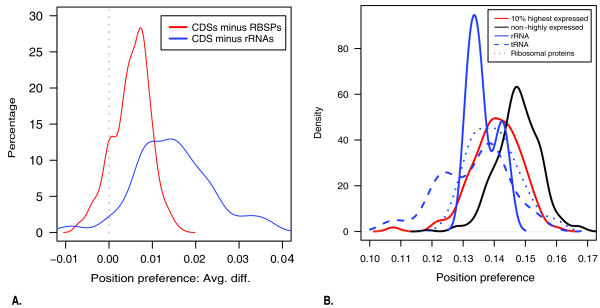
**Gene density plots**. (A) Density plot of position preference differences for 328 microbial genomes. Differences between mean position preference of translated coding sequences (CDSs) and ribosomal proteins (red) or between mean position preference of CDSs and ribosomal RNA (rRNA) (blue). Most microbial genomes CDSs have a higher mean position preference values than ribosomal proteins and rRNA (mean above 0). (B) Position preference densities for the 10% most highly expressed genes, non-highly expressed genes, rRNAs, tRNAs and ribosomal proteins in *E. coli*.

Examining the ribosomal proteins for *E. coli*, we confirm that the average position preference is lower than for other protein encoding genes (Wilcoxon P-value 4e-11), and it is even more extreme for non-translated genes. For example, rRNAs, tRNAs, and miscellaneous RNAs have significantly lower position preference values than translated genes (P-value = 6e-34). Although this difference was observed for a majority of the 328 microbial genomes examined, ribosomal proteins are not always encoded by DNA with low position preference (Figure 2A). However, the difference in position preference of ribosomal proteins and non-ribosomal proteins correlated very well with the replication times of the cells (P-value = 5.1e-10) using the number of 16S rRNAs as an indirect measure of doubling time, as previously suggested [20], since the number of 16S rRNAs indirectly influence replication times [21]. Thus, fast replicating microbes demonstrated a larger difference in position preference between ribosomal proteins and non-ribosomal proteins than slow replicating microbes. Consequently, as for CAI [6], fast replicating microbes have optimized their translational machinery by increasing the expression of proteins such as ribosomal proteins. As a result, their expression is optimized both by codon usage and by placing them in regions where DNA is easily accessible (Wilcoxon P-value = 5e-10).

While highly expressed ribosomal proteins and non-translated RNA genes demonstrated a tendency to have low position preference especially in fast replicating organisms, this does not signify that the position preference measure for prediction of highly expressed genes may only work in fast replicating organisms as for the CAI measure. On the contrary, position preference might, consequently, provide an alternative measure for prediction of highly expressed genes in slow replicating bacteria. For these, ribosomal proteins and RNA genes are not always highly expressed and therefore, the CAI measure is less efficient.

The above results are somewhat in contrast to the findings by Segal and coworkers [22]. They recently published a more refined model for nucleosomal positioning based on a combined experimental and computational approach. Although this model predicts a nucleosome pattern strikingly similar to that of the model used in our study [13,22], at least for eukaryotes, they did not find nucleosome depletion at ribosomal proteins sites in Yeast. Consequently, they predicted high nucleosome occupancy encoded over these genes and reasoned that the expression of these genes is governed by other factors. However, although we only predict a slightly lower than average position preference for yeast ribosomal proteins, we find that the general trend observed across a large range of microbial genomes is that both DNA encoding ribosomal proteins and non-coding genes have lower position preference than the genomic average (Figure 2). This points at a possible regulation of ribosomal proteins by DNA structural properties.

### Position preference versus CAI

The position preference measure used in this study is based on the experimentally determined preference demonstrated by individual trinucleotides to be positioned in a specific orientation in nucleosomal DNA [13]. Consequently, the position preference score assigned to any given triplet will be the same for all organisms and the gene average will depend only on the specific sequence of a gene whereas CAI scores for a gene depends on both the sequence and the translational codon bias in the specific organism to which the gene belongs. Correspondingly, we only found small correlations or anti-correlations between CAI triplet weights and position preference triplet scores for a few organisms, none of which were significant (multiple testing [23] corrected *P*-values = 1). Moreover, the correlation between CAI weights and position preference triplet values did not increase for fast replicating bacteria (*P*-value = 0.532), indicating that position preference as such, may be a useful supplement for predicting highly expressed non-translated genes even in slow-growing microbes. Moreover, because rRNAs, tRNAs, and other non-coding RNAs tend to have lower position preference than the genomic average, the position preference measure could be useful for identifying these genes in pre-annotated DNA sequences. In particular, because the position preference can be estimated at the DNA level and as such, do not require the prior knowledge of gene co-ordinates.

### Prediction of highly expressed genes

From Figure 1, we would expect a correlation between low position preference and high gene expression level. However, a complete separation of highly expressed genes from the other genes was not possible using the position preference measure (for example, see Figure 2B). This is hardly surprising since no structural or coding property singularly determines the level of gene expression, for which a large number of regulatory steps are involved. Consequently, the level of separation may reflect the influence of each measure on gene expression. For the five additional microbial genomes where we had experimentally determined expression values, a clear difference was also observed between the distributions of CAI or position preference values for highly expressed genes and low expressed genes. For details, refer to supplementary Table S1 [Additional file 1].

**Figure 3:**
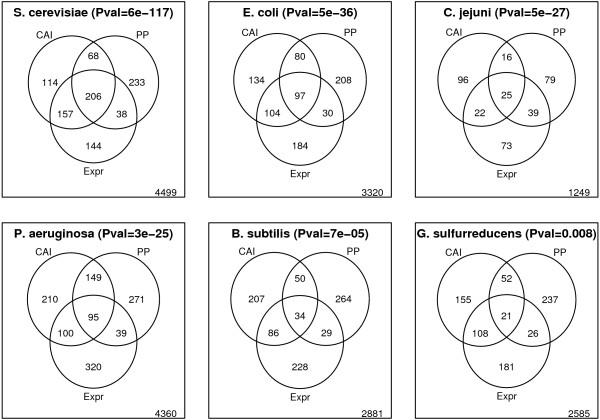
**Venn diagram**. Illustrates the overlap between genes predicted to be highly expressed by CAI (top 10%) and position preference (bottom 10%), and genes with high microarray gene expression values (top 10%). Thus, the number found in the intersection between all three circles refer to the number of genes predicted to be highly expressed by either measure and also found to be highly expressed experimentally. The number found outside the circles refer to the number of genes that are not highly expressed and not predicted as such by either method. The organisms are ordered by the significance of the overlap between position preference (PP) and highly expressed genes (Fisher's exact test [40]).

As expected from the above analyses, we observe a significant enrichment in highly expressed genes among genes with low position preference (Figure 3) for all 6 organisms for which we have microarray gene expression data available. Moreover, the correlation between position preference values and microarray gene expression values is highly significant [see supplementary Table S2, additional file 1]. However, the overlap between genes with high CAI values and highly expressed genes is even more significant (Figure 3). While this is expected since codon usage is known to have a strong influence on protein expression, the DNA structural properties also influence gene expression, and it seems reasonable that DNA which cannot be condensed into tightly wrapped chromatin structures is more accessible to RNA polymerase, which is about the same size as a nucleosome. One likely explanation is that position preference, as a measure of chromatin structure, might not be the most optimal – particularly for bacterial genomes. This might also explain the considerably higher enrichment in highly expressed genes among *S. cerevisiae *genes with low position preference than observed for the bacterial genomes (Figure 3).

While CAI values are better predictors of high expression of proteins, DNA structural properties may be used for prediction of gene expression for non-translated genes such as transfer RNAs and micro RNAs. For example, for *E. coli*, gene expression levels were further available for some non-translated genes. Including these in the comparison, the overlap between genes with low position preference values and genes with high expression values were more significant (p-value: 3.6e-45) than when only including translated genes in the comparison (p-value: 5.0e-36). Table 1 lists the 55 non-translated genes predicted to be highly expressed by the position preference measure. More than 70 percent of these are also found to be highly expressed experimentally. Both of these findings demonstrate that not only may the position preference measure be used for predicting the gene expression level for non-coding regions, but since these regions are even more correlated with anisotropic DNA flexibility than translated genes, they may consequently be under even more strict regulation by DNA structural properties. This makes sense because regulation by codon usage obviously makes no sense for transcripts that do not code for proteins.

### Functional categories of genes with low position preference

**Figure 4:**
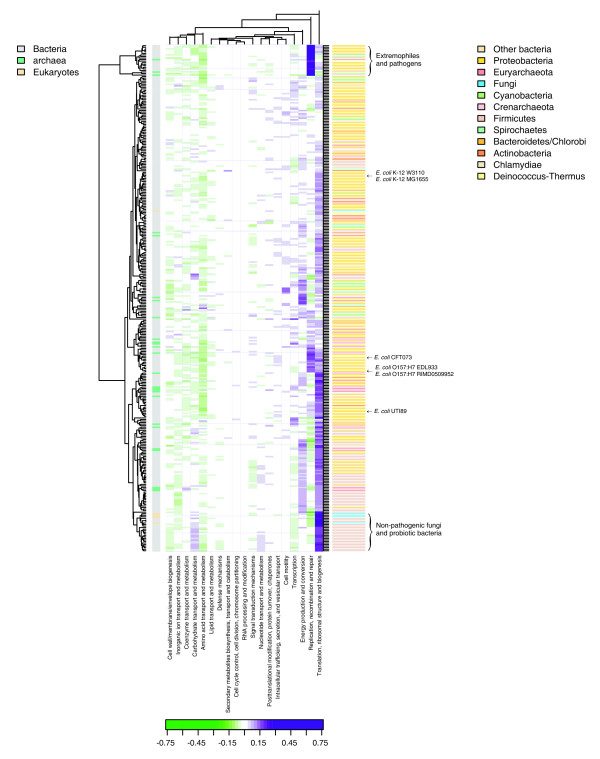
**Heatmap of COG functional categories for genes with low position preference (10% lowest) for 328 microbial genomes compared to the genomic background**. The color scale goes from -0.75 to 0.75 and represents the frequency of occurrences in each COG category among genes with low PP minus the overall frequency of occurrences in the genome. Over-represented categories among genes with lowest PP compared to the genomic background is indicated with purple, while green indicates under representation. For example, if all genes with low position preference corresponded to a certain COG category, and the same category only existed in 25 percent of all genes in that organism, the score would be 0.75 for that cog category for that organism. The kingdom is indicated as a vertical color bar to the left between the heatmap and the dendrogram. The phyla is illustrated as a vertical color bar to the right.

In fast growing organisms, ribosomal proteins and other proteins involved in translation and transcription are often highly expressed and are extremely biased in their codon usage preferences, that is, they have high CAI values [5]. Genes involved in translation, transcription, replication, and energy production are often encoded by anisotropically flexible DNA in terms of low position preference values which is thought to be correlated with high gene expression (Figure 4). Figure 4 (and supplementary Figure S1 [additional file 1]) illustrates over-represented (purple) and under-represented (green) COG functional categories among genes with low position preference relative to the genomic background. The COG categories and the microbes are clustered in two dimensions by hierarchical clustering and the microbes do not cluster according to AT content (data not shown) as we found when clustering based on codon usage bias [6]. Instead, it is possible to see the COG categories of genes encoded by DNA with low position preference. For most microbes, DNA with low position preference encodes genes involved in 'translation, ribosomal structure and biogenesis', 'energy production and conversion', 'transcription', and various types of metabolism.

It is clear that the clustering brings together organisms which are relatively distant phylogenetically (Figure 4), right side color bar representing the taxonomic phylum of each genome). As opposed to the apparent clustering according to similar environments as found based on CAI [6], in the present analysis, the ordering appeared related to the functionality of the microbe, i.e. pathogen versus non pathogen. For example, the COG category 'replication, recombination and repair' is particularly over represented amongst genes with low position preference for a distinct cluster at the top of Figure 4, consisting of extremophilic archaea and bacteria as well as pathogenic bacteria (mainly *Yersinia pestis *and *Shigella *strains). The common feature of these organisms is that genes involved in replication, recombination and repair have very low position preference (and consequently are potentially highly expressed). Particularly genes involved in recombination and repair are essential for pathogens and microbes living under extreme conditions making it reasonable for them to be highly expressed. Supporting this observation, we find that the same COG category is over represented for pathogenic *E. coli *strains, O157:H7 EDL933, O157:H7 RIMD0509952, CFT073 and UTI89 as well as for most *Shigella *strains, which are essentially pathogenic *E. coli*, whereas, the same COG category is not dominating for the non-pathogenic *E. coli *strains K-12 W3110 and K-12 MG1655. This provides us with a possible means for distinguishing pathogenic strains from non pathogenic strains. An important caveat is that some pathogenic strains have important virulence genes expressed on plasmids, which were not considered in this study. The more direct approach to distinguishing pathogenic strains from non-pathogenic strains is to look for pathogenicity factors. However, the exact combination of virulence genes and pathogenecity factors necessary to make a strain pathogenic is still unknown and also depends on the expression level of these genes.

Finally, four fungi clustered closely with certain probiotic bacteria (*Lactobacillus*); it is interesting to note that these organisms can live in a similar ecological niche. Also, a few microbes contain genes with low position preference that are involved in carbohydrate transport and metabolism, especially the *Streptococcus *genomes found in the bottom cluster of Figure 4. Again, this might be reflective of their ecological niche.

The above analysis demonstrates that the overrepresented COG categories differ between microorganisms independently of phylogeny. Moreover, the differences in the occurrences within the 'translation, ribosomal structure and biogenesis' COG category may explain why the position preference measure was more effective in some organisms than others according to Figure 3. Consequently, instead of the above speculation that position preference is an eukaryotic measure and therefore works better in *S. cerevisiae *than in bacteria, the very high representation of this COG category among genes with low position preference in *S. cerevisiae *could explain why position preference is a better predictor of gene expression levels in *S. cerevisiae *than in prokaryotes, in particular *G. sulfurreducens *where this COG category is barely present among genes with low position preference.

## Conclusion

We use a nucleosome position preference measure of anisotropic DNA flexibility to predict highly expressed genes in microbial genomes, and compare it to a translational codon adaptation index for synonymous codon usage bias of potentially highly expressed genes. We hereby demonstrate that absolute gene expression levels are highly correlated with low position preference in multiple microbial genomes. This newly gained insight into DNA structure dependent gene expression may be exploited for predicting the expression of non-translated genes such as non-coding RNAs that may not be predicted by any of the conventional codon usage bias approaches, and we speculate that it may also be used for prediction of highly expressed genes in slow growing microbes, in which the CAI measure is less successful. Genes often encoded by DNA with low position preference values were mostly involved in 'translation, ribosomal structure and biogenesis', 'energy production and conversion', and transcription. For pathogens and microbes living in extreme environments, the predominant functional category was 'replication, recombination and repair'. In particular, *E. coli *pathogenic strains and most *Shigalla *strains demonstrated this trait while non pathogenic *E. coli *strains did not. This provides a likely signature for distinguishing some pathogenic strains from non pathogens. This new insight into DNA structural dependent gene expression in microbial genomes may aid in our understanding of gene expression regulation. It may also be used in developing a reliable predictor of gene expression both in prokaryotes and eukaryotes.

## Methods

### Translational Codon Adaptation Index (CAI)

The codon adaptation index describes a codon usage bias in an organism [4]. Here, we use a translational codon adaptation index (CAI), in which a codon bias signature is deduced that is most likely to be efficient for translation [6]. In short, this method is based on a known set of 27 very highly expressed *E. coli *genes for bacterial genomes [24], and a set of 39 very highly expressed yeast genes for eukaryotes [25]. Both reference sets were identified based on protein expressions. In order to identify a set of constitutively highly expressed genes for each of the bacterial genomes analyzed in this work, the reference set of very highly expressed *E. coli *or Yeast genes is aligned at the protein level against all genes annotated in the Genbank entry for each genome using BLASTP version 2.2.9 [7]. For each of these very highly expressed genes, the gene with the best alignment was added to a set of very highly expressed genes if it had an E-value below 10^-6^, and these were used as a reference set for the given organism. Using each genome specific reference set, a weight table including all codons is derived indicating the most translationally efficient codons. In turn, these weights are used for calculating a CAI value for each gene. The higher the CAI score, the more likely a gene is to be highly expressed.

### Position preference

This is a model of anisotropic DNA flexibility, which is derived experimentally from the preference demonstrated by individual trinucleotides to be positioned in a specific orientation in nucleosomal DNA [13]. The values indicate the preference of triplets for being specifically positioned in nucleosomal DNA. High absolute values correspond to triplets with a strong preference for having minor grooves facing either towards or away from the nucleosome core, while triplets with close-to-zero preference can occupy any rotational position on the nucleosomal DNA, and are thus assumed to be flexible in one direction. Since the 'position preference' measure is based on a simple trinucleotide model, values are assigned to every nucleotide in the DNA sequence simply by looking up the values for the corresponding triplet, in which the nucleotide is centered [1,14,15]. Here, the average of each possible triplet in a gene is used to calculate the position preference score for that gene.

### Assigning Cluster of Orthologous Genes (COGs)

The system for delineation of Clusters of Orthologous Groups of proteins (COGs) is based on orthologous relationships between genes and is useful for comparative genomics and facilitates the functional annotation of genomes. Here, genes were assigned a COG category by AutoFACTS, an automatic functional annotation tool [26] utilizing Blastx version 2.2.9 [7] to blast open reading frames to a database of sequences with assigned cog categories available from NCBI [27]. The following COG categories were not used due to their low relevance in microbial functional genomics: 'chromatin structure and dynamics' (B), 'nuclear structure' (N), 'cytoskeleton' (Z), and 'extracellular structures' (W). Also, the two categories of poorly characterized functions were neglected: 'general function prediction only' (R) and 'function unknown' (S).

### Prediction of ribosomal proteins

Ribosomal proteins for each Genbank entry were predicted using profile Hidden Markov Models (HMMs) from Pfam [28] since the quality of the annotations available from the Genbank entries varies tremendously. Pfam_ls profile HMMs for all ribosomal proteins were extracted (94 as per July 24^th ^2006). Pfam_ls files contain all the Pfam models for finding global or complete matches to a domain or family.

### Gene expression data

Microarray based gene expression data were taken from Willenbrock et al., 2006 [6]. Briefly, the dataset comprised pre-processed gene expression data for *E. coli *[29], *C. jejuni *[30], *P. aeruginosa*, *S. cerevisiae *[31,32], *G. sulfurreducens *[33], and *B. subtilis *[34]. Additional microarray gene expression data for *E. coli *at different growth stages were taken from [35], where raw data were normalized with qspline [36] and expression indices were estimated [37].

### Data treatment

All DNA and protein sequence information was extracted from each of the 328 Genbank entries. For correlation estimates, we used Spearman's rank correlation [38] to avoid any problems with possible deviations from normality in compared data (e.g. log-normal distribution for microarray data). Cluster analysis was based on hierarchical clustering of Euclidian distances using complete linkage. For density plots, the bandwidths were chosen as the standard deviation of the Gaussian smoothing kernel.

### Supplemental information

Additional data are available at our website [39]. This website contains an overview of the 328 microbial genomes included in this study linked to estimated position preference values. Supplementary Figure S1 is a detailed version of the heatmap sketched in Figure 4, providing the full organism names of all included microbial genomes. Supplementary table S1 and S2 provides some statistics for the comparison of expression values and CAI and position preference.

## Abbreviations

CAI: Codon adaptation index

PP: position preference

CDS: coding sequenge

COG: cluster of orthologous genes

HMM: Hidden Markov Models

## Authors' contributions

HW performed the analysis, interpreted the results and drafted the manuscript. DW participated in the design of the study and assisted in editing the manuscript. Both authors read and approved the final manuscript.

## Supplementary Material

Additional File 1 Supplementary. Supplementary data for 'Prediction of highly expressed genes in microbes based on chromatin accessibility'. Click here for file

## Figures and Tables

**Table 1:** Predicted highly expressed non-translated *E. coli *genes by the position preference measure.

*Gene*	*bnumber*	*Type*	*Gene expression rank*	*PP*		*Gene*	*bnumber*	*Type*	*Gene expression rank*	*PP*
		
**asnT**	**b1977**	**tRNA**	**122**	**0.1076**		**leuP**	**b4369**	**tRNA**	**404**	**0.1294**
**asnW**	**b1984**	**tRNA**	**121**	**0.1076**		selC	b3658	tRNA	617	0.1295
**asnU**	**b1986**	**tRNA**	**101**	**0.1076**		**thrT**	**b3979**	**tRNA**	**143**	**0.1300**
**asnV**	**b1989**	**tRNA**	**127**	**0.1076**		**serW**	**b0883**	**tRNA**	**196**	**0.1310**
thrV	b3273	tRNA	898	0.1154		**rrsH**	**b0201**	**Ribosomal and stable RNAs**	**15**	**0.1318**
valW	b1666	tRNA	1544	0.1172		**rrsA**	**b3851**	**Ribosomal and stable RNAs**	**1**	**0.1318**
**valT**	**b0744**	**tRNA**	**114**	**0.1212**		**tyrU**	**b3977**	**tRNA**	**172**	**0.1318**
**valZ**	**b0746**	**tRNA**	**130**	**0.1212**		**rrsG**	**b2591**	**Ribosomal and stable RNAs**	**6**	**0.1319**
**valX**	**b2402**	**tRNA**	**124**	**0.1212**		**rrsC**	**b3756**	**Ribosomal and stable RNAs**	**2**	**0.1320**
**valY**	**b2403**	**tRNA**	**109**	**0.1212**		**rnpB**	**b3123**	**misc_RNA**	**73**	**0.1332**
**aspU**	**b0206**	**tRNA**	**219**	**0.1215**		leuZ	b1909	tRNA	920	0.1338
**aspV**	**b0216**	**tRNA**	**233**	**0.1215**		**rrfF**	**b3272**	**Ribosomal and stable RNAs**	**14**	**0.1338**
**aspT**	**b3760**	**tRNA**	**150**	**0.1215**		**rrlH**	**b0204**	**Ribosomal and stable RNAs**	**4**	**0.1348**
serU	b1975	tRNA	1420	0.1221		**metU**	**b0666**	**tRNA**	**309**	**0.1351**
leuU	b3174	tRNA	3814	0.1229		**metT**	**b0673**	**tRNA**	**229**	**0.1351**
valV	b1665	tRNA	2022	0.1233		**rrlA**	**b3854**	**Ribosomal and stable RNAs**	**12**	**0.1351**
**thrU**	**b3976**	**tRNA**	**66**	**0.1235**		**rrlC**	**b3758**	**Ribosomal and stable RNAs**	**18**	**0.1352**
**ileU**	**b3277**	**tRNA**	**274**	**0.1239**		**rrlE**	**b4009**	**Ribosomal and stable RNAs**	**5**	**0.1352**
**ileT**	**b3852**	**tRNA**	**236**	**0.1239**		**rrlG**	**b2589**	**Ribosomal and stable RNAs**	**3**	**0.1354**
argQ	b2691	tRNA	455	0.1241		**rrlD**	**b3275**	**Ribosomal and stable RNAs**	**30**	**0.1355**
argZ	b2692	tRNA	765	0.1241		**lysT**	**b0743**	**tRNA**	**123**	**0.1357**
**argV**	**b2694**	**tRNA**	**401**	**0.1241**		**lysW**	**b0745**	**tRNA**	**142**	**0.1357**
ffs	b0455	misc_RNA	686	0.1255		**lysY**	**b0747**	**tRNA**	**94**	**0.1357**
trpT	b3761	tRNA	1483	0.1276		**lysZ**	**b0748**	**tRNA**	**131**	**0.1357**
pheV	b2967	tRNA	3047	0.1285		**lysQ**	**b0749**	**tRNA**	**113**	**0.1357**
pheU	b4134	tRNA	1995	0.1285		dicF	b1574	RNA; Cell division	3004	0.1361
**leuT**	**b3798**	**tRNA**	**171**	**0.1287**		proK	b3545	tRNA	3076	0.1367
leuQ	b4370	tRNA	502	0.1287						

These 55 non-translated genes are among the 10% genes with lowest PP values (listed from lowest to highest PP values). The 39 genes highlighted in bold are also found to be highly expressed according to microarray gene expression levels and their rank are shown according to the measured microarray gene expression level (from highest to lowest, low rank => highly expressed).
