# Supplementary material for: Prediction of highly expressed genes in microbes based on chromatin accessibility
Source: BMC Mol Biol. 2007 Feb 13;8:11. doi: 10.1186/1471-2199-8-11 (PMC1805505; doi:10.1186/1471-2199-8-11)
Supplement: Additional File 1 — Supplementary. Supplementary data for 'Prediction of highly expressed genes in microbes based on chromatin accessibility'. [file 1471-2199-8-11-S1.pdf]

Table S 1. Significance (P-values) of difference between tCAI or position preference values of genes with either 10% highest or 10% lowest expression values.

	tCAI	position preference
E. coli	~0	1.5e-34
P. aeruginosa	~0	8.4e-15
G. sulfurreducens	1.5e-14	3.2e-3
C. jejuni	~0	3.1e-28
B. subtilis	~0	2.5e-12
S. cerevisiae	~0	5.8e-97

Table S 2. Comparison of position preference values and microarray gene expression values.

	Spearman's correlation (rho)	overlap between 10% lowest PP and 10% most highly expressed, Fisher's exact test
S. cerevisiae	-0.36 (P-value = 2.7e-168)	5.9e-117
E. coli	-0.20 (P-value = 1.7e-39)	1.3e-37
P. aeruginosa	-0.20 (P-value =9.2e-51)	3.3e-25
G. sulfurreducens	-0.11 (P-value = 5.7e-11)	0.0083
C. jejuni	-0.44 (P-value = 6.2e-77)	5.1e-27
B. subtilis	-0.11 (P-value = 7.3e-11)	7.2e-05

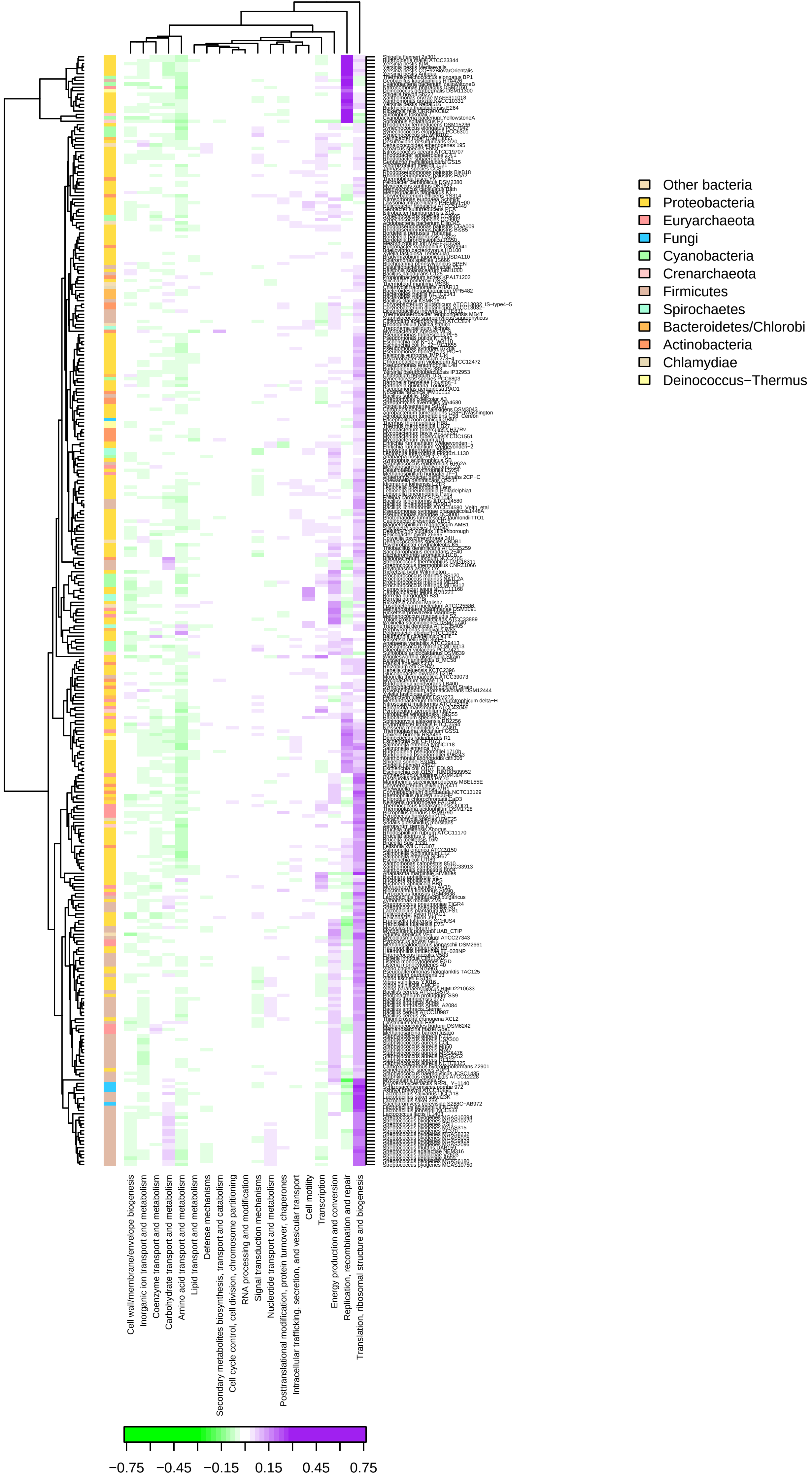

Other bacteria
Proteobacteria
Euryarchaeota
Fungi
Cyanobacteria
Crenarchaeota
Firmicutes
Spirochaetes
Bacteroidetes/Chlorobi
Actinobacteria
Chlamydiae
Deinococcus−Thermus
Cell wall/membrane/envelope biogenesis
Inorganic ion transport and metabolism
Coenzyme transport and metabolism
Carbohydrate transport and metabolism
Amino acid transport and metabolism
Lipid transport and metabolism
Defense mechanisms
Secondary metabolites biosynthesis, transport and catabolism
Cell cycle control, cell division, chromosome partitioning
RNA processing and modification
Signal transduction mechanisms
Nucleotide transport and metabolism
Posttranslational modification, protein turnover, chaperones
Intracellular trafficking, secretion, and vesicular transport
Cell motility
Transcription
Energy production and conversion
Replication, recombination and repair
Translation, ribosomal structure and biogenesis
−0.75
−0.45
−0.15
0.15
0.45
0.75
